# Existing ‘In‐Between’: A Qualitative Exploration of Individual, Interpersonal and Structural Influences on Empowerment Among Young Adults With Chronic Health Conditions

**DOI:** 10.1111/hex.70863

**Published:** 2026-10-19

**Authors:** Tiana A. Kittos, Danielle M. Muscat

**Affiliations:** ^1^ Graduate School of Health University of Technology Sydney Sydney New South Wales Australia; ^2^ Sydney Health Literacy Lab, Sydney School of Public Health, Faculty of Medicine and Health The University of Sydney Sydney New South Wales Australia

**Keywords:** chronic health conditions, empowerment, individual, interpersonal, qualitative, structural, young adults

## Abstract

**Background:**

Empowerment is a multifaceted construct conceptualised in different ways across the literature. Traditionally individual in its orientation, patient empowerment has more recently been acknowledged as also encompassing interpersonal and structural elements. As young adults with chronic health conditions enter the developmental stage of ‘emerging adulthood’ and transition from paediatric to adult care, empowerment may play a key role in supporting health management.

**Objective:**

This study aimed to explore what empowerment means to young adults, and the facilitators and barriers that co‐exist at an individual, interpersonal and structural level. Additional aims were to explore healthcare providers' perspectives and to inform how empowerment is framed in this context.

**Design:**

We adopted a qualitative study design, drawing on phenomenology as a theoretical orientation and research methodology.

**Setting and Participants:**

Twelve online semi‐structured interviews were conducted; six with young adults with chronic illness and/or disability, and six with healthcare providers.

**Analysis:**

Thematic analysis was used to inductively analyse the data with a deep emphasis on reflexivity.

**Results:**

Three themes were developed from the data: (1) Navigating healthcare in the ‘in‐between stage’ of life; (2) ‘I'm just adding to my tool kit’: Stories of self‐reflection, advocacy and knowledge gain on the pathway to empowerment; and (3) ‘A safe space’: Exploring how healthcare providers, interactions and spaces facilitate empowerment. The findings revealed that young adults with chronic health conditions encountered contradictory structural processes within healthcare systems that did not consistently equip them to self‐manage their health, despite increasing expectations for autonomy and responsibility. Nevertheless, many demonstrated considerable agency, proactively acquiring the knowledge, skills, resources, and support necessary to foster empowerment and navigate the demands of self‐managing a chronic health condition.

**Discussion:**

Our findings emphasise the co‐existence of individual, interpersonal and structural elements of empowerment, and the tensions that arise during the in‐between stage of young adulthood.

**Conclusions:**

This study highlights that young adults require unique support to facilitate empowerment, with both barriers and facilitators in this process. Research should continue to privilege the young adult voice to improve empowerment outcomes and healthcare experiences for young people.

**Lived Experience Contribution:**

T. A. K. acknowledges her contribution of dual expertise as a researcher and as a young adult with lived experience of cancer.

**Patient or Public Contribution:**

Young adults with varying chronic illnesses and/or disability were involved in the data collection stage of this project. These participants contributed their invaluable perspectives and insights through online interviews. Healthcare providers of varying professions were also involved in the data collection stage of this project, with their contributions being provided through an online interview. At the heart of this study are the voices of the participants.

Empowerment is a multifaceted construct conceptualised in different ways across the literature. In health, patient empowerment is traditionally defined as the process of activating a patient to change their behaviour and self‐manage their illness and health [1], as well as the changes to wellbeing and lifestyle that co‐occur [2]. A patient's perceived confidence in participating in consultations and managing their health condition independently, for example, may be seen as an indicator of an empowered patient [3].

Despite this individual orientation, patient empowerment is also discussed as encompassing interpersonal and structural elements. Here, the empowerment process is seen to intersect multiple structures at micro, meso and macro levels, spanning the patient, healthcare provider and broader healthcare system. Broader conceptual models of empowerment acknowledge the role of the healthcare provider in the exchange of information and resources about illness, which develops self‐efficacy and increased personal control for a patient [4, 5]. The clinician–patient relationship – specifically, the trust, support, and collaborative dynamic between them [6] – is essential and is integrated in this process. From a structural perspective, environmental structures can facilitate access to opportunities for skill development, information, informal and formal support, and resources [7, 8], which, in turn, can impact individual motivation, confidence and autonomy [9]. A systematic review investigating qualitative research on patient perspectives of empowerment similarly identified structural elements, in addition to individual and interpersonal aspects [10].

While overall, there is an extensive body of literature related to the concept of empowerment, research on patient empowerment in young adults is still growing. Young adults with chronic illness represent an increasing proportion of all young adults in Australia and globally [11]. Similarly, the population of young people aged 15 to 24 years old with a disability has increased from 9.4% in 2018 to 13.9% in 2022 [11]. This increase could be attributed to advances in the identification of disability and in medical treatments allowing children to survive into young adulthood [12], and emphasises the importance of the young adult demographic.

Young adults aged 18–29 years old enter a developmental stage of life known as ‘emerging adulthood’ [13]. This time of transition is marked by biopsychosocial changes and specific experiences relating to identity, instability, feeling ‘in‐between’, open possibilities (referring to friendships, relationships, and occupational opportunities) and increased self‐focus [14]. Additional challenges, such as an increased responsibility to obtain knowledge to self‐manage their health, less parental support, navigating the healthcare system and new financial considerations related to their chronic conditions [15] form part of their ‘emerging adulthood’. Their ‘transition’ point is often also characterised by a physical transitionary process where they move from paediatric to adult care services [16]. However, research has highlighted how young adults can often experience a lack of continued care [17], face health system complexity and fragmentation [18], access barriers [19] or transition without condition‐specific knowledge and information [15, 20]. Many face discrimination, particularly those experiencing multiple marginalisation [18]. Thus, the competing stressors of emerging adulthood may be amplified for young adults with chronic conditions.

Small et al. [5] identified five dimensions of empowerment in young people with chronic conditions, including identity, knowledge, personal control, decision‐making and supporting other patients [21]. Úcar Martínez et al. [22] further position the ‘empowered’ young person as someone who is active in seeking information, makes decisions about their care and enables their voice to be heard. The ‘empowered’ young person can take charge and improve their quality of life [22].

More recent empowerment literature has also focused on the role of the healthcare provider, describing the healthcare provider as needing to provide holistic care, promote the development of self‐management skills and facilitate the coordination of care for young people with chronic health conditions [23]. While empowerment as a structural experience has not been explicitly explored from the perspective of young adults with chronic health conditions, previous studies have similarly identified structural factors in the empowerment process for this demographic. For example, in a metasynthesis of qualitative literature, young people with cystic fibrosis identified that empowerment is facilitated by obtaining increased access to information, increased understanding of their condition, opportunities to develop confidence, access to supportive relationships and connections, as well as being listened to and feeling respected [24]. A systematic review investigating the transition to adult care similarly found that young people valued receiving more information about their condition and the transition process [25]. This sentiment was mirrored in a later systematic review focusing on facilitators of empowerment for cancer patients during follow‐up care, which revealed the importance of access to information and respect between the patient and healthcare provider [26]. Opportunity for support and group connections was also important [26]. This could suggest that access to increased knowledge, resources, opportunities and external supports is part of the empowerment process and can reveal the need to reframe part of empowerment as structural.

The aim of this research project was to explore the facilitators and barriers to empowering young adults with chronic health conditions and how they operate within the empowerment process. The current study explored the following research questions:

1. What are young people's perspectives on empowerment, including the facilitators and barriers to empowering young adults with chronic health conditions at individual, interpersonal and structural levels?
2. What are healthcare provider's perspectives on the facilitators and barriers to empowering young adults with chronic health conditions?
3. How can these facilitators and barriers inform how we frame empowerment?

## 1 Materials and Methods

### 1.1 Study Design

This study used an exploratory qualitative research design, involving semi‐structured interviews with young adults living with chronic illness and/or disability, as well as healthcare providers who have worked with young adults. The study drew on phenomenology as a theoretical orientation and research methodology. Phenomenology is concerned with understanding human experience in terms of ‘individual consciousness’ [27]. The empirical focus is on the individual experience, appropriate for our interest in privileging the voices of young adult and healthcare providers and their experiences in relation to empowerment. The reporting of this qualitative study follows the consolidated criteria for reporting qualitative research (COREQ) [28]. Ethical approval was obtained through the University of Technology Sydney Human Research Ethics Office (ref: ETH24‐9495).

### 1.2 Participants

Young adult participants were eligible to participate in this study if they were between the age of 18–25 years old, had a chronic illness and/or disability and spoke and understood English. We explored 18–25 as a specific age range within 'emerging adulthood' [1, 13] due to it's relevance to the transitionary point of moving from paediatric to adult healthcare services. Healthcare provider participants were eligible to participate in this study if they had experience working with young people with chronic illness. Throughout this paper, the terms *young adults* and *young people*, have been used interchangeably to reflect the language of the participants in this study. Similarly, *illness, disability, chronic condition* and *condition*, have been used interchangeably to reflect the diverse health experiences and language used by the participants in our study.

### 1.3 Sampling and Recruitment

A convenience sampling method was used to recruit participants [29]. Participants were recruited from an external organisation's mailing list, known as Child UnLimited. Child UnLimited is a national network of young people, parents, clinicians, and researchers committed to improving the quality of life of young people with chronic illness and disability [30]. A flyer was distributed via email to the Child UnLimited mailing list (which includes 110 researchers and clinicians, 55 consumers and 25 not‐for‐profit organisations) outlining the study and participation requirements. Snowball sampling, where participants share the study with other individuals [31], also naturally occurred as per the ethics protocol.

From the initial invitation email, participants were prompted to complete a Qualtrics online expression‐of‐interest form and were assessed for eligibility against the inclusion criteria. Eligible participants reviewed the participant information sheet and completed the consent form and thereafter filled a demographic questionnaire to collect personal data. They were then contacted to organise an interview.

Interviews were conducted online via Zoom by both D. M. M. and T. A. K., who are trained in qualitative methods. Interviews lasted 45 min on average (range: 30–71 min). All interviews were audio‐recorded and transcribed verbatim using the Zoom transcription function. These transcripts were checked with the recording to confirm accuracy and facilitate data familiarisation. The data was analysed concurrently with data collection to assess theoretical saturation. Theoretical saturation was pragmatically considered using its original conceptualisation; the point at which gathering more data about a theoretical construct reveals no new properties and the construct is deemed saturated [32]. Pseudonyms were used to maintain the anonymity of participants and to better reflect the individuality and humanity of participants. This approach aligns with the interpretive aims of qualitative research, which seek to understand and represent participants as individuals situated within particular social and experiential contexts, while still protecting their identities and privacy.

### 1.4 Materials

#### 1.4.1 Demographic Characteristics

Age, gender identification, postcode, highest level of education, language spoken at home, chronic illness and/or disability, years since transitioning to adult care, and health literacy (measured using the Single Item Literacy Screener [33]) were collected for the young adult participants. Healthcare provider demographic data included: age, gender identification, clinical role, health specialisation and duration working in that area with young people.

#### 1.4.2 Interview Schedule

The interviews in this study were semi‐structured, using pre‐determined open‐ended questions [34]. Two semi‐structured interview schedules were developed (see Appendices A and B) with modified language to suit each participant sample, and kept general to promote flexibility and allow participant responses to drive the discussion. Definitions of empowerment were not provided in the interviews, and the questions followed from how the participants defined empowerment. This was deemed more appropriate, as this study aimed to maintain authentic discussions to privilege the unique experiences and understanding of our participants. However, questions that sought an understanding and perspective on structural empowerment were informed by Kanter's [7] theory of structural empowerment as well as the work of Laschinger et al. [8].

### 1.5 Participant Demographics

In total, 12 people participated in a semi‐structured interview: 6 young adults with varying chronic illnesses and/or disability and 6 healthcare providers. All 12 participants were included in the data analysis. The demographic information of the young adult participants is presented in Table 1. All young adult participants were within the age range of 18–25 years (mean age = 22.4, SD = 2.3), and the majority identified as female. All (100%) young adult participants had completed high school and a university degree, further education or vocational training. All participants had adequate health literacy, as per the Single Item Literacy Screener.

**Table 1:** Young adult participant demographic information.

	Number of participants	Percentage of participants
Age range		
18–25	6	100%
Gender^b^		
Female	4	67%
Male	1	17%
Not disclosed	1	17%
Highest level of education^b^		
University degree, further education or vocational training	5	83%
Not disclosed	1	17%
Language spoken at home		
English	6	100%
Confidence in completing medical forms^b^		
Quite a bit	2	33%
Extremely	3	50%
Not disclosed	1	17%
Chronic illness and/or disability^a^, ^b^		
Vertebral defects, anal atresia, cardiac defects, tracheoesophageal fistula, renal and limb anomalies (VACTERL)	1	17%
Attention‐deficit hyperactivity disorder	1	17%
Automatic neuropathy	1	17%
Intestinal failure	1	17%
Gastroparesis	1	17%
Cerebral palsy	1	17%
Obsessive‐compulsive disorder	1	17%
Generalised anxiety disorder	1	17%
Not disclosed	1	17%
Number of years since transitioning to adult care^b^		
2–3	1	17%
4–5	2	33%
5–6	1	17%
6+	1	17%
Not disclosed	1	17%

^a^ Total exceeds the number of participants as some young people had multiple chronic conditions and/or disabilities. Total also exceeds the amounts represented in the other demographic data presented within this table. The percentage of participants also reflects this.

^b^ Demographic data missing for *n* = 1.

The demographic information of the healthcare provider participants is displayed in Table 2. Healthcare provider participants were aged between 30 and 60 years (mean age = 45.7, SD = 8.6) and were female. One participant was a medical doctor, two were clinical nurse consultants, and the other three participants were psychologists. Most participants had worked with young adults for 6 years or more, and the majority (*n* = 5) worked in oncology.

**Table 2:** Healthcare provider participant demographic information.

	Number of participants	Percentage of participants
Age range		
30s–40s	2	33%
40s–50s	2	33%
50s–60s	2	33%
Gender		
Female	6	100%
Clinical role		
Psychologist	3	50%
Medical doctor	1	17%
Clinical nurse consultant	2	33%
Chronic illness area		
Cancer	5	83%
Varying chronic conditions	1	17%
Years working with young people with chronic illness		
2–3	1	17%
5–6	1	17%
6+	4	67%

### 1.6 Data Analysis

Thematic analysis was used to analyse the data. Thematic analysis is defined as an approach that searches for relations within data and describes them to then create themes [35]. This analysis used an essentialist perspective and an inductive approach, focusing on the reality of participant experiences and the emerging data to drive the project, rather than producing meaning outside of the individual [35]. This analysis was informed by the six phases outlined in Braun and Clarke [35]. See Table 3.

**Table 3:** Steps of thematic analysis outlined in [35], as applied to this study.

1. Familiarisation	**Phase 1** involved T. A. K. familiarising herself with the data through transcription, data checking and re‐reading all transcripts. This familiarisation stage afforded the opportunity to begin interpreting the data for meaning.
2. Generating initial codes	**Phase 2** involved the generation of initial codes within the data. T. A. K. read each individual line of the transcript and attached a code to it. The codes in this study were data‐driven, meaning themes were not derived from theory. Coding was an effective way to group data and identify patterns. As recommended, T. A. K. coded any possible idea resulting from the data in the case of future relevance [35]. D. M. M. on this project checked a purposefully selected sample of codes to ensure that accurate codes were attached to represent the data collected.
3. Searching for themes	**Phase 3** began the stage of searching for themes. Codes were organised into broader ideas about the data, known as themes, using Microsoft Word. Themes were identified at a semantic level, meaning each focused on description and experience, as opposed to underlying assumptions that inform the data. This stage also involved the search for possible sub‐themes to help see patterns across the data. A thematic map was created to help visualise these relationships amongst the data.
4. Reviewing themes	**Phase 4** involved conducting a review of the themes to see if there was sufficient evidence for each. T. A. K. considered Welch and Patton's [36] criteria, which looks at internal homogeneity and external heterogeneity, assessing whether themes collaborated meaningfully whilst also being different from one another. During this phase, T. A. K. reviewed the codes for each possible theme to decide whether a pattern formed and updated and reconstructed the thematic map to reflect changes as the analysis progressed.
5. Defining and naming themes	**Phase 5** involved providing a definition and name for the chosen themes. T. A. K. reviewed the themes, chose and explained the interesting features of the data from participants to creatively illustrate the essence of the themes. This stage further involved reviewing sub‐themes to ensure they fit appropriately within the theme and across the whole story of the data. During this phase, D. M. M. and T. A. K. collaborated and compared interpretations to verify themes and sub‐themes. This was important in practising reflexivity, as ‘the reflexive researcher acknowledges that any finding is the product of the researcher's interpretation’ [37, p. 45].
6. Production of the report	**Phase 6** involved writing up the results and confirming that there was sufficient evidence to support each theme and form a coherent, flowing story that represented the experiences and perspectives of the participants. T. A. K. at first was more descriptive about the data; however, she made several revisions to integrate an argument about the facilitators and barriers to empowering young adults with chronic health conditions. As D. M. M. conducted multiple interviews and reviewed coding, she had a strong familiarity with the data, which assisted in ensuring that T. A. K.'s personal experiences with cancer and empowerment did not drive the story of this data.

### 1.7 Reflexivity

Wilkinson [38] outlines interrelated personal, professional, and disciplinary layers of reflexivity. This framing informed how reflexivity was thought about in the current study and the approaches practised to address the researchers' positionality across all project stages. From conceptualisation, T. A. K. considered her understanding of empowerment and motivations that directed her to decide on this research enquiry [39] as being shaped by her experience as a cancer survivor. Her research interest was informed by her regular engagement with others with lived experience and her academic knowledge as a psychology student, including existing psychological theories and qualitative research in this area [39]. Bracketing – defined as a process of uncovering and acknowledging preconceived knowledge and beliefs about a research topic [37] – was achieved through research diaries, a practice interview, and iterative discussions with D. M. M. This increased T. A. K.'s self‐awareness and allowed her to recognise how her personal, professional and disciplinary perspectives were informing decisions such as the framing of questions in the interview guides [37].

During the interviews, our team decided that T. A. K. would not disclose her lived experience as a cancer survivor to participants to ensure the focus of the interviews was participant experiences. We also centre the direct quotes of participants in our reporting. Memoing was also used as a strategy to maintain methodological rigour and promote self‐awareness within data interpretation [37]. A decision‐trail was included within memoing diaries during the initial stages of the research process to explain decisions and to track understanding [37], and memoing notes were discussed during data analysis meetings. All steps of thematic analysis included both a researcher with lived experience (T. A. K.) as well as a researcher (D. M. M.) without lived experience, with a focus on discussing differing interpretations of the data.

## 2 Results

Three themes were constructed through our thematic analysis: (1) Navigating healthcare in the ‘in‐between stage’ of life, (2) ‘I'm just adding to my tool kit’: Stories of self‐reflection, advocacy and knowledge gain on the pathway to empowerment and (3) ‘A safe space’: Exploring how healthcare providers, interactions and spaces facilitate empowerment. Across all themes, there were seven subthemes.

### 2.1 Theme 1: Navigating Healthcare in the ‘In‐Between Stage’ of Life

Participants expressed that young adulthood is an undefined category ‘where you're not a child, but you're not an adult’ (Alex, missing data for age). Experiencing illness and navigating healthcare as a young adult in this ‘in between age’ (Grace, healthcare provider, oncology) was described as being ‘so different to an older person’ (Lucy, healthcare provider, oncology). Healthcare providers highlighted the need to identify the care of young adults ‘as a unique subspeciality’ (Jean, healthcare provider, oncology).

#### 2.1.1 Theme 1.1: Liminality, Invisibility and Dismissal

The young people that we interviewed shared how ‘specialists and doctors … can be very quick to dismiss your concerns’ (Gina, 24) as a young adult. Some young people felt that there was a need to be sick enough to access care, particularly in general practice (GP) or primary healthcare settings, to avoid being dismissed by healthcare providers. One young person felt the need to create ‘self‐imposed rules’ such as waiting for ‘three issues before [they] present to the GP's office, because [they] couldn't ever present with just one’. This was also described as a form of validation to mitigate feeling like a burden to the healthcare system, ‘wasting time for [themselves] or for others’ (Alex, missing data for age) as a young adult. Despite such strategies, young adults still reported experiences of feeling dismissed when engaging with health services.

It was sort of dismissed as like growing pains or being oversensitive, and stuff like that. So, like, you know when you're told that you trust the doctors, and you're like, ‘Oh, [it] just must be me. This is how everyone must be feeling’.

(Melody, 19)

Such challenges and feelings were amplified for young adults with an invisible illness who reported that they felt the need for young adults to be in a critical condition to engage with healthcare.

Doctors often just do not treat patients until they're … like, I joke about it like almost dead, but … it's come to a point where so many people are just not presenting until they're at that point of like, barely conscious, because … doctors are not treating patients unless they're anything but that. It's not all doctors, but it's [a] very big problem within our health system.

(Angelique, 22)

I think it's the struggle of invisible illnesses and people not understanding … just cause I'm not like screaming or saying that I'm tired doesn't mean I'm not in pain, and I'm not tired.

(Melody, 19)

#### 2.1.2 Theme 1.2: The Tactical Compromise of Independence

In a context where the majority of young people expressed ‘not being taken [seriously]’ and feeling ‘dismissed’ (Melody, 19) by health staff, they reported making tactical choices to build credibility. Many, for example, reported having a parent present during consultations in order to be believed.

I tend to go … with my … parents, because it tends to be easier to be believed when there's someone else backing you up where, when it's just yourself … like even really simple things like I went, and I was like, ‘hey, could you fill my script?’ And they were like, ‘I'm not doing that’.

(Melody, 19)

Although parental presence was deemed necessary and sometimes helpful (e.g., for relaying medical history), it came into tension with young people's expressed desires to have the ‘freedom to be able to make [their] own choices’ (Matt, 25). That is, it was perceived as a barrier to feeling empowered for young people who shared that their independence is threatened when they ‘have to bring [their] parents along everywhere’ (Angelique, 22).

I will not present without my parents there, which makes it hard … I feel less empowered having them there, I would say, because I feel like I'm just not being believed or like I don't know. They're … fantastic support, but I think it's not a good thing having to rely on them.

(Angelique, 22)

One young person felt the need to adopt strategies to gain a sense of control in such consultations, such as removing the opportunity for their parent to be acknowledged first.

When my mum's there they used to just talk to her and then direct their questions towards me, while now I immediately start talking so they don't have the opportunity to turn to mum and go ‘Okay, so what do you think about this?’.

(Alex, missing data for age)

#### 2.1.3 Theme 1.3: ‘Left in the Dark’

Young people needing to access adult care services felt that there was a notable difference in support for adults compared to young adults.

You find there's a lot of support for adults who pass the age of young adults. But there's not really much for these people that are between the age of probably like 18 and 25 because you kind of don't fit in a box.

(Angelique, 22)

In this context, healthcare providers pointed out that feelings of powerlessness are often amplified for young people, a sentiment mirrored by the young adults in our study who described a healthcare system and healthcare providers who ‘kind of push you to the side’ (Alex, missing data for age). Transitioning between paediatric to adult services is where young adults felt this most particularly, especially in contexts where young adults reported receiving no follow‐up to connect services or to support their transition between care services.

We were left with no one. We were told someone existed, but we would never put in contact. We were never given their number. We were never given an email so we don't know who it is, and I still don't know who it is.

(Alex, missing data for age)

### 2.2 Theme 2: ‘I'm Just Adding to My Tool Kit’: Stories of Self‐Reflection, Advocacy and Knowledge Gain on the Pathway to Empowerment

Despite young people and healthcare providers expressing that ‘knowledge is power’, a majority of the young people reported having gaps in their knowledge and could not recall ‘any [resources] that [were] provided’ (Melody, 19) to them to manage their condition. A lack of knowledge and support was associated with feeling powerless. One young person shared that during in‐patient care, they were not equipped to manage their illness, making it a ‘very disempowering environment’ (Ella, 22).

I remember coming out of that experience feeling very different and feeling so unequipped to continue to deal with the problems I was dealing with because I hadn't really been taught anything. So, I think definitely after that I just did have a moment of feeling … like I was pretty powerless.

(Ella, 22)

#### 2.2.1 Theme 2.1: Looking Inwards and Investigating Outwards

Many young participants used an inward focus to make sense of their chronic health condition, which allowed them to be a source of information for themselves. One young person leaned into their awareness of being a ‘visual learner’ and attended webinars to understand their own symptoms.

So I like the webinars [and] attend a whole bunch of different webinars, and I'm like my symptoms match up with that. Let's present that to a doctor.

(Melody, 19)

One young person found it ‘empowering for [them] to actually self‐reflect and see how much [they've] grown’ in adversity, using it to ‘build … resilience’ (Ella, 22).

When I go through a really rough period … I'll write it down so that I can reflect on it later … because when I fall into another kind of period of being overwhelmed or stressed or down … I forget that I've done it before … and so, having things like that is really important to help me kind of get out of those periods of struggle.

(Ella, 22)

Similarly, for another young person who was experiencing depression and did not have familial support, using self‐reflection as a skill allowed them to enact change for themselves, emphasising the power of the young person.

I made the conscious effort to actually open myself up, begin exploring my emotions, begin exploring what I wanted out of life, begin exploring what my troubles were, what I liked about myself and my family life … what I didn't like about myself.

(Matt, 25)

Some young people also conducted independent research to gain clarity around their symptoms and diagnosis and to better understand their condition. One young person felt this was helpful as healthcare providers were perceived to sometimes lack the necessary knowledge or time to thoroughly explore or explain their health concerns, specifically for rare conditions. Another young person relied on medical textbooks to gain relevant knowledge. Knowing how to safely navigate research was identified as an important skill to have, particularly when the internet was identified as not always ‘being a reputable source’ (Alex, missing data for age).

[I turned] to Google and ended up finding my condition, which I'm now diagnosed with … Now I do a ton of research and follow the foundations and the societies and I'm just constantly reading research papers and different things to try and pull together, cause when you get diagnosed with the condition a lot of the time … they put all your issues down to this one thing but there can actually be things underlying that and if you can manage that, then your function and quality of life, and the things you're able to participate [in] and do significantly increase. But they don't do a deep enough dive, or are knowledgeable, in the first place to actually look at the mechanisms.

(Melody, 19)

…I now own medical textbooks that have portions of my condition in it. I have access to websites and the knowledge of how to research medical conditions.

(Alex, missing data for age)

#### 2.2.2 Theme 2.2: Leveraging Social Connections and External Support

Young people shared how they used external avenues to obtain knowledge, skills and support to manage their health. Utilising the experiences of other people with illness and/or disability was also identified as a resource to obtain knowledge. Social media groups were a way to learn ‘tips and tricks’ to assist with daily functioning.

Reading the Facebook pages and Instagram of people that shared their lived experiences, I saw what my symptoms were, and I also learned tips and tricks through their management … I trial different things, and a lot of them worked, and a lot of them didn't but I'm just adding to … my toolkit.

(Melody, 19)

Connecting with others who share similar experiences also offered a ‘different level of understanding’ (Ella, 22) that transcended connections with those without health conditions.

If you're in a group of people there's just this … unspoken thing sort of like bond that goes beyond friendship. It's different. I don't know how to explain it.

(Melody, 19)

I don't know how to explain this … [there's] just a different level of understanding, talking to someone else about my experience … rather than talking to someone who doesn't.

(Ella, 22)

For the young people who have required continued support during young adulthood, one way of upskilling themselves was through self‐enroling in peer support programmes.

One young person felt they had not only gained knowledge but also purpose through advocating with others with chronic health conditions, acquiring a feeling of empowerment gained through ‘representation and community’ (Melody, 19).

I got to work alongside other people with chronic health conditions and disabilities, and how vocal they were … that kind of inspired me.

(Alex, missing data for age)

Through connecting with others and through advocacy and all the skills I've learned. I feel like it's really empowering to know that it's sort of given me a purpose and something I'm really passionate about.

(Melody, 19)

### 2.3 Theme 3: ‘A Safe Space’: Exploring How Healthcare Providers, Interactions and Spaces Facilitate Empowerment

Healthcare providers were considered to play a role in empowerment and took intentional interpersonal and structural steps to facilitate empowerment for young people. Both healthcare providers and young people recognised the importance of ‘getting to know the young person’ (Grace, healthcare provider, oncology) as a first step towards empowerment. One young person expressed how important it is to be spoken to ‘like they're a human being’ (Alex, missing data for age). Four young people shared this sentiment, suggesting that safe and empowering spaces were facilitated by a healthcare provider that will ‘just listen’.

Empowerment is having a doctor or nurse or any health care provider really, that will sit and listen, and that won't judge you.

(Angelique, 22)

#### 2.3.1 Theme 3.1 ‘Knowing That You Have a Choice’

All young people associated freedom and choice with feeling empowered. One young person particularly noted that empowerment was related to being ‘given [the] choice of numerous pathways to go down to … actually accomplish a goal, or just to be myself in general’ (Matt, 25).

Such freedom and choice were valued especially within the context of consultations with healthcare providers. Here, young people expressed the importance of knowing about their choices, but also having multiple options. One participant associated a healthcare provider giving them the opportunity to review different treatment options and making time to discuss these options, without being ‘forced into doing anything’ (Angelique, 22), as empowering.

She printed me off all of the research I needed for these 3 different things, and she was like, ‘I want you to take time to go away and think about it’ … it was more like a human interaction. … she was very respectful in saying ‘there's no judgment what way you go. I really want you to look at these, and then come back and talk to me, and if these aren't good enough, or if you don't feel comfortable, we can think of something else.’ … That was a moment where I feel empowered. … I did have a say, and I wasn't gonna be told that [I was] non‐compliant.

(Angelique, 22)

In a mental health context, one young person spoke of the importance of being aware of the choice to try different healthcare professionals until finding one that supports you. Finding the right one was considered ‘life‐changing’ (Ella, 22), contributing to the participant's feelings of empowerment.

Knowing that you have a choice to kind of go see a different psychologist, if you want to … do a bit of shopping if you have to, I think that like that's really important as well.

(Ella, 22)

Healthcare was not the only space important to young people to have choices and options. For example, one young person shared that ‘knowing your options … is really empowering’ (Melody, 19). This young person shared that the need for options extends to a school setting, as young people living with chronic illness and/or disability can ‘feel really stuck’ (Melody 19).

There's other options. You can do distance education. You can do TAFE digital. You can do all these other things, but no one talks about them, because everyone's pushing you down the steam train.

(Melody, 19)

#### 2.3.2 Theme 3.2: ‘A Space at the Table’

In order to create a safe space, healthcare providers spoke of the need for young people to firstly have ‘a space at the table … where decisions are being made … [and be] part of conversations’ (Celeste, healthcare provider, oncology). However, the presence of this space was neither certain nor assured. The participants we spoke to acknowledged there was not always enough space for young people to be able to have input into their care and wants.

It's that revolving door of the doctor coming in, the nurse coming in, health professionals coming in and saying this is what you need to do. … From my perspective, it felt like there wasn't a lot of space for the young person to say or to even have input.

(Lucy, healthcare provider, oncology)

Consequently, participants discussed the need for healthcare professionals to play an active role in building such a space for young people that ensures they ‘get the information [so] that…, [they are] able to take part in decision making’ (Diana, healthcare provider, oncology) as a source of empowerment.

We need to create an environment where young people feel safe with your help and it feels safe enough to get out certain information … personalised about their health.

(Jaimie, healthcare provider, broad range of chronic illness)

So for us it's to ensure that … their voices are … listened to, and empowered.

(Jean, healthcare provider, oncology)

Part of creating this space involved challenging certain power imbalances that exist between the healthcare provider and young person. Another young person reflected on how they wish to shift the power as a future healthcare provider.

I think there was a bit of a power imbalance … I was obviously a very young woman, and this is like [a] much older and much louder and sort of, you know, scarier professional.

(Gina, 24)

I think about how would I want to interact with my patients when I'm a doctor. I think that plays a big role as well like wanting to have a focus on patient advocacy rather than … ‘Oh, I'm the doctor … I make all the decisions’.

(Angelique, 22)

Another important way to make space for the young person involved is to acknowledge that the young person has a unique and essential level of expertise about their health.

You are also an expert sitting here in this room because you're the expert on you. Like, you're the only [one] who knows what it's like to walk in your shoes and to go through what you're going through.

(Diana, healthcare provider, oncology)

## 3 Discussion

This qualitative exploration of empowerment with 6 young people and 6 healthcare providers working with young people continues the extensive work in the empowerment space. However, to our knowledge, it is the first Australian study to investigate facilitators and barriers existing on an individual, interpersonal and structural level from the perspectives of young adults and healthcare providers. This study revealed that young adults exist within an ‘in‐between’ developmental space where there is an expectation of increased autonomy, responsibility and self‐management – however, they encounter contradictory structural processes within healthcare that do not always equip them to manage this change. There are expectations to embody new developmental responsibilities; however, a lack of space to do so. Despite the structural and interpersonal barriers that operate within the empowerment process, our study also highlighted how young adults themselves curate their own empowerment and actively disrupt the structures that coexist with them.

The current study echoed previous research exploring the lack of transition support to adult services and continuity of care, with the common trajectory of ‘slipping through the cracks’ [15, 17, 40]. The challenges existing between childhood and adulthood [17] and the medical invalidation faced by young people with chronic health conditions [41, 42] were also endorsed by our study. However, this current work crucially highlights a tension between the growing independence that is both expected in, and characterises, young adulthood [13, 43]. It demonstrates that there is a need to produce credibility (through building a portfolio of health issues or through parental presence at appointments), as merely being a young person presenting with health concerns is not enough to access support independently.

Our study also highlights the resourcefulness of the young person as a beacon of knowledge and facilitator of empowerment. Young people in the current study actioned their own opportunities to build knowledge about health management and to upskill their advocacy abilities to navigate healthcare interactions and conditions more confidently. Connecting with others with shared experience also facilitated empowerment, reinforcing prior research [26] and serving multiple purposes, including providing self‐management tips [44] and validation [45].

Our research endorsed the importance of healthcare providers using a patient‐centred communication approach [46] and practising shared decision‐making [47]. Specifically, collaboration, support [6], being listened to and respected [24] are essential for young people to make informed choices. However, our study offers a different lens to view this facilitator – structural empowerment, whereby power is part of the process in facilitating access to information and skills [8] to manage chronic health conditions. The current study highlights how healthcare providers grapple with their own interpersonal and structural conditions to create a space at the table for the young person's expertise.

### 3.1 Clinical Implications and Future Recommendations

The findings of this study offer insight into the tensions of being a young adult with a chronic health condition and the unique support required during this developmental period. Future research could work towards a co‐produced model of empowerment for young adults that integrates and intentionally acknowledges the barriers during this process, and the facilitators identified by young people and healthcare providers at all levels: individual, interpersonal and structural.

With a concrete theoretical model, future research could also seek to empower young people through participatory design opportunities [48] to co‐produce a toolkit for healthcare providers and health systems to refer to when supporting this age group.

### 3.2 Strengths and Limitations

The strength of the current study lies in the in‐depth thematic analysis, the triangulation of the young adult and healthcare provider voices, and the reflexive practices flowing through T. A. K.'s consumer–researcher voice [37]. However, our sample (both young people and healthcare providers) was primarily recruited from Child UnLimited. This may have influenced the findings as participants were connected to an advocacy organisation and may therefore have had greater access to support, information, and opportunities to develop self‐advocacy skills. While participants still described challenges advocating for themselves within the healthcare system, these difficulties may be even more pronounced among young adults who are not connected to advocacy or support organisations, or who face multiple marginalisations [18]. Additionally, we acknowledge that the young people in our sample all spoke English, had pursued further education and had adequate health literacy. This undoubtedly shaped their knowledge, help‐seeking and healthcare experiences, which were shared through the interviews. Studies highlight that socially marginalised young people may have differing experiences of healthcare, often culminating in a strong distrust of services [49], which would necessarily inform their conceptualisation of empowerment. Similarly, healthcare providers in our study all identified as female and self‐selected to participate in a study about empowerment in young people. Consequently, the findings may overrepresent perspectives that value and prioritise empowerment and may not fully capture the views and practices of healthcare providers who are less engaged with this concept.

A future consideration will be to better understand ways to engage diverse young people in research to strengthen our exploration, ensuring that the engagement is mutually fulfilling and that appropriate renumeration is provided for time committed [50].

## 4 Conclusion

This study reframes patient empowerment as an interactive process whereby individual, interpersonal and structural empowerment co‐exist. The facilitators identified emphasise what empowerment can mean to young adults, but the barriers acknowledge the tensions embedded within becoming an ‘empowered young person’. There is a need to recognise the issues persisting in transition support within the healthcare system and the crucial role of healthcare providers to work with young adults to equip them with the knowledge, skills, resources and support to manage their health confidently. However, the most important learning of this study is the power and resourcefulness of the young person, and this encourages future research to continue to privilege the voices of this age group.

## Author Contributions

**Tiana A. Kittos:** writing – original draft, writing – review and editing, conceptualisation, investigation, methodology, validation, data curation, project administration, formal analysis. **Danielle M. Muscat:** writing – review and editing, supervision, resources, project administration, conceptualisation, formal analysis, validation.

## Funding

The authors have nothing to report.

## Ethics Statement

Ethical approval was obtained through the University of Technology Sydney Human Research Ethics Office (ref: ETH24‐9495).

## Consent

All participants provided written informed consent to participate in this study.

## Conflicts of Interest

Danielle M. Muscat receives personal royalties from the sale of a book on health literacy, *Health Literacy in a Nutshell*. She is also the co‐director of *Health Literacy Solutions PTY Ltd.*, which sub‐licences a health literacy editing tool. She takes no personal income from this role. The other author declares no conflicts of interest.

## Data Availability

The data that support the findings of this study are available from the corresponding author upon reasonable request.

## Appendix A

### A.1

**Interview questions – Young adult**

[Introductory text]

1. There is lots of discussion about empowerment when it comes to young people in care – What does this term mean to you/how does it resonate with your experience?
  a. Prompt – Has this changed over time?
2. Can you tell me about a time when you have felt empowered or disempowered in regard to your health?
  a. Prompt – What do you think facilitates empowerment for a young person?
  b. Prompt – What do you think is a barrier to young people feeling empowerment?
3. Can you reflect on how important each of the following are for empowerment:
  a. Access to increased knowledge
    i. What kind of information do young people need to have to manage their illness?
      ▪ Prompt – Does this resonate with your knowledge? What type of information has been helpful and unhelpful?
  b. Opportunities to grow confidence or skills.
    i. What kind of skills do young people need to manage their illness?
  c. Increased resources
    i. What kind of resources do you think are important to young adults navigating health/and young adulthood?
      ▪ Prompt – Does this resonate with your experience? What type of resources have you been offered?
  d. Social structures
    ▪ What kind of support do young people need to manage their illness/young adulthood?
      a. Prompt – Healthcare providers, family, friends, school, and so forth
      b. What do you think healthcare providers need to know to support a young person during this time?
  e. Self‐determination
    i. Did you feel that you had choice and freedom in decision‐making?
    ii. Impact/influence – Did you think that you could impact or influence decisions about your health?

## Appendix B

### B.1

**Interview questions – Healthcare provider**

1. How do you support young people with chronic illness in your care?
2. What does empowerment for young people mean to you/look like to you as a healthcare provider?
3. Using that definition that you provided me, tell me about a time where you have felt that you empowered a young person?
4. What do you think is a barrier to empowering a young person in this way?
5. Is there anything about empowering a young person that you wish you knew more about/don't understand too well?
6. Do you feel that you have been equipped to know how to empower a young person? Why/why not?
7. Can you reflect on how important each of the following are for empowerment:
  a. Access to increased knowledge
    i. What kind of information do health care providers need to have to help young people manage their illness?
      1. Prompt – Does this resonate with your experience?
  b. Opportunities to grow confidence or skills.
    i. What kind of skills do young people need to manage their illness?
      1. Who should help young people gain these skills?
      2. In your experience, have you felt equipped to help young people develop these skills?
  c. Increased resources
    i. What kind of resources do you think are important to young adults navigating health/and young adulthood?
      1. Prompt – Does this resonate with your experience? What type of resources have been available for you to provide?
  d. Social structures
    1. What kind of support do young people need to manage their illness/young adulthood?
      a. Prompt – Healthcare providers, family, friends, school, and so forth
      b. What do you think healthcare providers need to know to support a young person during this time?
  e. Self‐determination
    i. How important do you think it is for young people to have choice and freedom in decision‐making about their health?
    ii. Impact/influence – Do/did you think young people need to feel like they can impact or influence decisions?
      1. How do you think healthcare providers can do this?
